# Alterations of Dendritic Cells in Sepsis: Featured Role in Immunoparalysis

**DOI:** 10.1155/2015/903720

**Published:** 2015-03-02

**Authors:** Xia Fan, Zheng Liu, He Jin, Jun Yan, Hua-ping Liang

**Affiliations:** State Key Laboratory of Trauma, Burns and Combined Injury, Research Institute of Surgery, Daping Hospital, The Third Military Medical University, Chongqing 400042, China

## Abstract

Sepsis, the leading cause of mortality in intensive care unit, is characterized by hyperinflammatory response in the early stage and followed by a period of immunosuppression. This immune disorder is believed to be the potent factor that is tightly associated with high mortality in sepsis. Dendritic cells (DCs) serve as professional antigen-presenting cells that play a vital role in immune response by activating T lymphocytes. During the progression of sepsis, DCs have been reported to take part in the aberrant immune response and be necessary for survival. Therefore, a better understanding of the DCs pathology will be undoubtedly beneficial for resolving the problems occurring in sepsis. This review discusses effects of sepsis on DCs number and function, including surface molecules expression, cytokines secretion, and T cell activation, and the underlying mechanism as well as some potential therapeutic strategies.

## 1. Introduction

Sepsis is high lethal public disease. In 2012, over 20 million people are affected by sepsis worldwide [1]. The mortality from septic shock and severe sepsis both in Europe and in USA is around 30% and this value is still elevated [1, 2]. Recently, sepsis is defined as the systemic inflammatory response syndrome (SIRS) due to infection [3], which indicated that SIRS and infection are two important factors in determination of sepsis.

When the host receives an infection, both pro- and anti-inflammatory responses are initiated. The inflammatory response is partly mediated by innate immune cells through recognition with invading pathogens or microorganisms [4]. These cells can decide the trend of inflammatory response toward pro- or anti-inflammatory state by producing proinflammatory cytokines (interleukin- (IL-) 1*β*, tumor necrosis factor- (TNF-) *α*, and interferon- (IFN-) *γ*) or anti-inflammatory cytokines (interleukin- (IL-) 10, transforming growth factor- (TGF-) *β*) [5, 6]. At the early stage of sepsis, there is a large amount of proinflammatory mediators termed as cytokines storm in the host. Therefore, various therapeutic methods have been used to treat sepsis by downregulation of proinflammatory cytokines expression. But in fact it does not bring good news in the clinical setting. There is one possibility that the animal model, such as cecal ligation and puncture (CLP), cannot entirely reflect the real state of septic patients, in which the gender, hormone, age, and other interference factors cannot be neglected [7, 8]. Another possibility is correlated with sepsis progression. Observation from clinical studies showed that about 80% septic patients had a persistence of infectious focus at the day they died [9]. Some other studies also found that the active cytomegalovirus normally existed in the septic patient without resolution [10, 11]. These results indicate that the host immunity exhibits a tolerance status, which makes the patients at an increased risk of subjection to secondary pathogen infection. The immunosuppression is found to be accompanied with immune cells deactivation and apoptosis, impaired antigen-presentation, suppression of proliferation of lymphocytes, and high levels of anti-inflammatory cytokines (IL-10). Moreover, polarization of T helper (Th) cells is toward to the Th2 type that results in an increase in susceptibility to infection. The aberrant immune response will further lead to multiple organ failure and death.

Among the innate immune cells, dendritic cells (DCs), firstly discovered by Raplph in the early 1970s, are the most potent antigen-presenting cells and central component for linking the innate and adaptive immunity [12–14]. DCs originate from bone marrow CD34^+^ stem cells and home to all tissues via the blood stream where they developed into immature cells [15]. Immature DCs have high phagocytic properties and readily take up antigen and present the antigen to Th cells. In response to endogenous danger signals or microbial antigens, DCs mature and migrate into the T cell area of lymphoid tissues, where CD4^+^ T cell will be activated. During the maturation, the phagocytic receptor will be lost, the surface molecules (e.g., MHCI, MHCII, CD80, and CD86) involved DCs migration, and T cells activation will be upregulated [16, 17]. Although many different classification manners have been described, two major subsets of DCs are recognized: myeloid DCs (mDCs) and plasmacytic DCs (pDCs) [18, 19]. The former is derived from bone marrow precursor and the latter is believed to evolve from circulating lymphoid precursor [20, 21]. These two types of DCs have a similar molecular phenotype except for CD8*α* ^+^, which is present in pDCs but absent in mDCs [22]. Based upon the importance of DCs in immune system and its central role in sepsis [23], this review will focus on the pathology changes of DCs during the evolution of sepsis.

## 2. The Effect of Sepsis on DCs Numbers

At first, large amounts of studies on animals or patients had featured obvious loss of CD4^+^ and CD8^+^ T cells in sepsis [24–27]. Due to the importance of DCs in the immune system, more and more investigators have focused on the change of DC numbers and its role in depletion of T cells. In general, CD11c is believed to be the common marker of murine DC for its steady state. A profound loss in the number of CD11c^+^ DCs was observed in spleen after sepsis and the time ranging from 12 h to 3 d [28–32]. When the CD11c^+^ DCs are further divided into CD8^+^CD4^−^, CD8^−^CD4^+^, and CD8^−^CD4^−^, it is found that CD8^+^CD4^−^ and CD8^−^CD4^+^ subsets were lost 36 h after CLP, but the number of CD8^−^CD4^−^ DCs was increased [33]. Thus it could be demonstrated that the reduced number of splenic DCs was mediated by a selective loss of CD8^+^CD4^−^ and CD8^−^CD4^+^ subtypes.

In addition to spleen, sepsis was also found to reduce the percentage of CD11c^+^ DCs present in local mesenteric nodes beginning 12 h after CLP and reach a 50% decline by 24 h. This phenomenon was also observed in systemic inguinal nodes, but not in popliteal nodes [34]. Moreover, another study was performed on the mice with CLP, which were subsequently intravenously challenged with* Schistosoma mansoni* eggs to develop granulomas. Results showed that there was a significant loss of DC in lung during the granulomatous response [35]. However, it should be noted that gradual reconstitution of DC numbers was found on postsepsis day 28 [30].

In clinical settings, the number of DCs in blood was lower in severe septic or septic shock patients in comparison with healthy controls [36, 37]. For two distinct populations of DCs, mDCs and pDCs, their numbers was markedly reduced in patients with sepsis when compared with controls, and both cell counts recovered slightly until day 28 [38]. But data from another clinical study of twenty-six patients showed that decreased mDC and increased pDC were observed at day 1, and the number of mDCs was not different in survivors and nonsurvivors of septic patients, while pDCs were obviously higher in nonsurvivors [39]. This discrepancy between these two study groups may be due to the different severity of illness. Moreover, reduction of circulating DCs can become a predictive factor for the development of septic complication after pancreatectomy [40]. Besides the adult patients, flow cytometric assay showed that the levels of pDCs and mDCs were also significantly lower in pediatric patients with sepsis [41].

**Figure 1:**
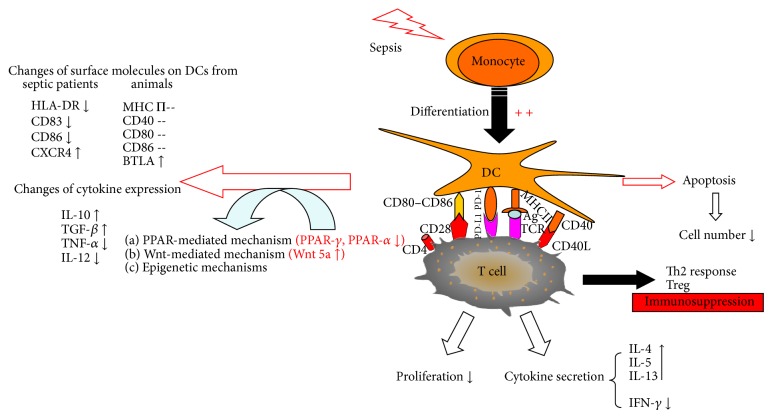
The changes of DCs during sepsis. When suffering from sepsis, DCs will be lost resulting from apoptosis, but differentiation from monocytes is accelerated. The surface molecules associated with DCs function are changed. At the same time, DCs have an aberrant cytokine secretion which results in immune tolerance status. The potential mechanism may be associated with apoptosis, PPARs, Wnt signal, and epigenetic regulation. MHCII: major histocompatibility complex class II, Ag: antigen, TCR: T cell receptor, PD-1: programmed cell death-1, PD-L1: programmed cell death ligand 1, BTLA: B and T lymphocyte attenuator, and PPARs: peroxisome proliferator-activated receptors.

In conclusion, sepsis causes the loss of DCs occurring in various lymphoid and nonlymphoid tissues from septic patients and septic mice. This phenomenon does not result from the inhibition of de novo generation of DCs from progenitors [42, 43], although these monocytic progenitors display characteristics of immunosuppressive properties [44] (Figure 1).

## 3. The Effect of Sepsis on DCs Function

### 3.1. Surface Molecular Expression

Upon the stimulation of microbial antigens or danger signals, DCs rapidly mature and migrate through the lymphatic system to lymphoid organs to stimulate T cells mediated immunity response. During this process, DCs will upregulate the presentation of cell surface proteins involved in T cell priming, including MHC, CD40, CD80, and CD86. In the CLP model, no obvious changes of CD40, CD80, and CD86 expression were discovered in CD11c^+^ splenocytes when compared with control group by 24 h after surgery. Similarly, peritoneal DCs showed CD40 and CD80 did not change in addition to an increase trend in CD86 expression [28]. However, splenic DCs from another study showed that levels of CD40 and CD86 were obviously enhanced by 15 h and 36 h after CLP while MHCI expression was much higher than control at 36 h following CLP. Only slight changes were observed in the expression of CD80 and MHCII [33]. For the DCs in lymph nodes, the percentage of CD40, CD80, CD86, and MHCII did not differ within 24 h between CLP-operated mice and sham-operated mice, but there was a much higher expression of these molecules 36 h after sepsis [33, 34]. In addition, sepsis did not cause the change of CD40 and CD80 in the lung until 7 d after CLP [45]. B and T lymphocyte attenuator (BTLA), a coinhibitory receptor, has been demonstrated to inhibit T cell activation and thus contributed to many diseases [46]. BTLA and its primary ligand, herpes virus entry mediator (HVEM), expressions were found to increase in immature and mature DCs in peritoneum by 24 h after CLP, while HVEM^+^ DCs were significantly decreased in bone marrow [47].

Clinical evidences proposed that the expression of human leukocyte antigen-DR (HLA-DR) is an indicator of immune failure, and with predictive value in clinical practice [48]. A profound decreased expression of HLA-DR on monocytes has been reported in septic patients [49]. But a continuous recovery phenomenon was exhibited in survivors of sepsis within 10 days, whereas there are no changes in nonsurvivors of sepsis [50, 51]. HLA-DR on mDCs in sepsis is three times lower than that in controls (MFI: 174 ± 54 versus 497 ± 128). Similar reduction was seen in pDCs, but with a narrower margin (MFI: 177 ± 66 versus 239 ± 77). At day 28, the expression of HLA-DR on mDCs was recovered but remained lower than that in controls, while HLA-DR on pDCs showed a similar expression pattern to controls [38]. Besides HLA-DR, the percentage of CD83 and CD86 was also reported to be reduced in septic patients, but chemokine receptor CXCR4 was upregulated [39].

### 3.2. Cytokine Secretion

A large number of studies have reported that septic DCs exhibit an aberrant cytokine secretion pattern, in which levels of proinflammatory cytokines (TNF-*α*, IL-1*β*, and IL-12) are significantly depressed and anti-inflammatory cytokines (TGF-*β*, IL-10) are enhanced [33, 38, 45] (Figure 1). DC-derived IL-12 is believed to be a key host defense cytokine and it is a heterodimeric cytokine composed of an IL-12p40 and IL-12 p35 subunit [30, 52]. Flow cytometric analysis of splenic DCs from LPS-primed mice revealed that the percentage of DCs able to produce IL-12 p40 was dramatically decreased from 1.7% to 0.3% [53]. When DCs were stimulated with TLR2 agonist (Pam3Cys) or TLR4 agonist (LPS) or TLR9 agonist (CpG-DNA), mRNA levels of both* Il12 p40 *and* Il12p35* from sepsis splenic DCs were significantly lower than that from sham splenic DCs [30]. Sepsis also resulted in a lower intracellular expression of IL-12 p40 induced by CpG-DNA compared with sham group [33]. In addition, only a small amount of IL-12 p70 was secreted from DC being stimulated with CpG or LPS + CD40L [33]. A similar trend was also seen in lung DCs. The DCs from lungs of postseptic mice with developing granulomas had a lower IL-12 p40 mRNA and IL-12 p70 protein levels compared with controls [35]. Moreover, they also exhibited defective IL-12 synthesis after TLR agonist challenge [45].

IL-10 is a pleiotropic cytokine possessing both anti-inflammatory and immunosuppression properties [54]. In the acute phase of sepsis, endogenous IL-10 production and exogenous administration can reduce the magnitude of the inflammation. Therefore, injection of recombinant adenovirus expressing IL-10, which limits DC maturation and associated T cell activation, could attenuate acute sepsis [55, 56]. However, the upregulation of IL-10 will result in the immunity tolerance that fails to defend the secondary pathogen challenge. 36 h after CLP, DCs from septic mice produced increasing amounts of IL-10 [33]. Upon incubation with TLR agonist, the higher level of IL-10 at both of mRNA and protein level was observed in splenic and lung DCs from postseptic mice in contrast to control [30, 35, 45]. The increased concentration of IL-10 in blood from septic patients is associated with worsened clinical outcome [57]. Furthermore, endogenous IL-10 has been reported to regulate IL-12 synthesis of DCs in an autocrine manner [58, 59]. DCs from sham mice could increase LPS-induced IL-12 expression in the presence of anti-IL-10 antibody. However, blocking of IL-10 could not rescue the production of IL-12 of postseptic DCs, which suggests that the low production of IL-12 during sepsis is not dependent on IL-10 expression [30].

### 3.3. T Cell-Stimulatory Capacity

The impact of DCs on T cells proliferation during sepsis was determined in a mixed leucocyte reaction (MLR). IL-2 plays a crucial role in the proliferation of T cells. It was found that the percentage of IL-2-secreting T cells was significantly lower when cultured with DC from septic mice as compared with control mice [33]. This finding was also confirmed when OT-II CD4^+^ T cells were incubated with DCs in the presence of antigen [60]. However, peritoneal DCs and splenic DCs from CLP mice both showed higher capacity to trigger proliferative response of T cells than those from sham group [28]. In addition, an increased activation of CD3^+^CD4^+^ T cell was also seen in the inguinal nodes and popliteal lymph nodes [34]. For septic patients, immature DCs from patients and health donors had a similar ability to induce T cells proliferation, but mature DCs from patients did not enhance T cell response [43].

Studies on polarization of T cells had showed that OVA peptide-specific CD4^+^ T cells secreted markedly higher levels of Th2 cytokines such as IL-5, IL-13, and IL-4 but a lower amount of Th1 cytokine IFN-*γ* when cocultured with postseptic splenic DCs that pulsed with OVA, indicating that Ag-loaded DCs direct T cells toward a Th2-dependent response during severe sepsis [30]. This is consistent with another study in which adoptive transfer of bone-marrow derived DC from septic mice impaired Th1 priming [42]. In addition, the expression of Foxp3 in T cells cocultured with patient or control DCs suggested that CD1a^+^ DCs from septic patients made the T cells have a stronger regulatory function, because the percentage of naïve T cells expressing Foxp3 when cultured in patient DCs was much higher than that induced by control DCs (93% versus 40%) [61], which suggested that sepsis led to an increase in regulatory T cells (Tregs).

In short, though controversy still exist, DCs will engender apoptotic or anergic T cells after sepsis. These anergic T cells, in turn, may disrupt DCs function.

## 4. The Potential Mechanisms Involving Changes of DC during Sepsis

### 4.1. Apoptosis-Dependent Mechanism

Studies by numerous groups have suggested that apoptotic death of immune cells plays a vital role in contributing to the immune hyporesponsiveness and organ injury during sepsis [62–64]. 24 h after CLP, a significant increase of apoptotic and dead DCs was found in mesenteric and inguinal nodes through the staining of annexin V [34]. This result was also confirmed by immunohistochemical staining for active caspase 3, a crucial mediator of apoptosis [29]. However, a high false-positive result may occur, because DCs have phagocytic properties and the positive signal may form the apoptotic debris that is phagocytized by DCs [65, 66]. To further clarify the relationship between apoptosis and the loss of DC, study from the transgenic mice which could overexpress the Bcl-2 reported that overexpression of Bcl-2 could dispel sepsis-induced DCs depletion. Furthermore, Bim^−/−^ mice exhibited remarkably less sepsis-induced loss in the DCs population [67]. Thus these proapoptotic and antiapoptotic proteins play a central role in DC loss during sepsis. In addition to DC loss, uptake of apoptotic DC would make viable DC display tolerogenic state that induces generation of Foxp3^+^ Treg [68].

The mechanisms by which sepsis caused DC apoptosis are at present not fully explored. A previous study has found that mechanism of apoptosis induced by LPS required activation of acid sphingomyelinase (A-SMase). Inhibition of this enzyme activity and ceramide generation could prevent apoptosis induction [69]. Furthermore, mammalian toll-like receptors (TLR)-dependent pathway is also found to involve in the process of sepsis-induced apoptosis, which was confirmed by several studies: (i) apoptosis of spleen DCs from CLP performed on TLR4^−/−^, TLR2^−/−^, and TLR2^−/−^ TLR4^−/−^ was inhibited [31]. (ii) TNF-*α*, a production of stimulation of TLRs, could impair mitochondrial integrity and induce apoptosis [70]. (iii) Interferon regulatory factor-1 (IRF-1) whose activation is dependent on intact TLR4 signaling was reported to trigger immune cells apoptosis [71]. However, a recent study showed that LPS-induced activation of nuclear factor of activated T cells (NFAT) via CD14 is necessary for DCs apoptosis, which was independent of TLR4 engagement [72].

### 4.2. Peroxisome Proliferator-Activated Receptors-Mediated Mechanism

Peroxisome proliferator-activated receptors (PPARs) are a superfamily of ligand-activated nuclear transcription factors and are involved in the regulation of lipid metabolism, glucose homeostasis, and cellular differentiation [73–75]. So far, three subtypes have been identified in human: PPAR-*α*, *β*(*δ*), and *γ*. Peripheral blood monocytes express high levels of PPAR-*α* and PPAR-*β* with low expression of PPAR-*γ* [76]. During the generation of DCs from monocytes and its maturation, PPAR-*γ* becomes the abundant subtype while the levels of other two subtypes are below the detection limit [76]. It was found that activation of PPAR-*γ* significantly increased the surface expression of CD36 and CD86 on LPS- and CD40 ligand-challenged DCs, whereas the synthesis of CD80, CXCL10, and CCL5 was reduced [77]. Moreover, it could depress the production of IL-12 with no effect on expression of IL-1*β*, TNF-*α*, IL-6, and IL-10 [77]. Studies also showed that PPAR-*γ* activation inhibited TNF-*α* induced DC migration from epithelia and subsequent accumulation in the draining lymph nodes [78]. Adoptive transfer of PPAR-*γ*-activated Ag-presenting DCs resulted in the impaired production of Th1 and Th2 cytokines, so as to induce CD4^+^ T cell anergy which fail to expand the secondary clone upon restimulation [79]. More interestingly, PPAR-*γ* was reported to be restricted to CD1a^−^ cells in the process of cytokine-induced DC differentiation. PPAR-*γ* transcriptional activity was higher in CD1a^−^ cells but not in CD1a^+^, indicating that the generation of CD1a^−^ cells might be associated with PPAR-*γ* [80]. However, a large number of CD1a^−^ cells were generated from peripheral blood monocytes of septic patients and the percentage of this type cells reached 68% after 7 d [61]. So it is not difficult to hypothesize whether the changes of DC in progression of sepsis were correlated to PPAR-*γ*. But there is no paper to clarify the connection between PPAR-*γ* and DCs in sepsis. Hepatic PPAR-*γ* mRNA expression and protein levels were reported to decrease at 20 h after CLP [81], but the results from another study showed that PPAR-*γ* expression of peritoneal cells was elevated significantly at both gene and protein levels 6 h after CLP [82]. Additionally, PPAR-*γ* expression in peripheral blood mononuclear cells from children patient with septic shock was also decreased but its activity was increased when compared to controls [83]. PPAR-*γ* activation could also promote T cell apoptosis in sepsis [84, 85]. Besides PPAR-*γ*, PPAR-*α* expression was reduced in patients with septic shock which was correlated to severity of illness [86]. Cell surface markers and cytokines production were decreased in PPAR-*α* knockout mice [86]. These data indicate the absence of PPAR-*α* is not beneficial for treating sepsis.

### 4.3. Wnt Signal Pathway-Mediated Mechanism

Wnt family is a highly conserved secreted signaling pathway that regulates developmental and homeostatic processes [87, 88]. Wnt proteins activate canonical or noncanonical signal pathway in a context-dependent manner [89, 90]. The former primarily takes part in cell fate determination and the latter is responsible primarily for cell movement and tissue polarity [91]. Wnt and their receptors are found to be expressed in hematopoietic progenitor cells (HPCs) [92], indicating that Wnt may be involved in HPCs differentiation. There was a remarkable expansion of hematopoietic cells after activation if Wnt canonical pathway. Wnt signaling pathway plays a central role in DCs differentiation in means of promotion on conventional DCs differentiation and inhibition on pDCs differentiation [93]. During the differentiation process of DCs from HPCs in vitro, Wnt signaling was upregulated characterized by accumulation of *β*-catenin and upregulation of Wnt target gene expression [94]. Activation of Wnt canonical pathway by Wnt 3a could promote the degeneration of CD11c^+^ DCs and enhance their capacity to stimulate T cells proliferation [94]. However, the activation of noncanonical Wnt pathway by Wnt 5a was shown to inhibit DC differentiation [94]. Wnt 5a-treated DCs had worse ability of capturing antigen. Wnt 5a had no effect on LPS-induced DC maturation but impaired the production IL-12p70 and TNF-*α* while increasing levels of IL-10. Furthermore, Wnt 5a inhibited the T cell proliferation and fail to prime T cell response [95]. So the two types of signal pathway display an opposite effect and sustain the regulation of DCs differentiation by crosstalking to each other. During sepsis, Wnt 5a concentration in sera of patients was elevated and Wnt 5a was also found to induce macrophage differentiation to a tolerogenic phenotype, which was related to induction of IL-10 and suppression of NF-*κ*B signaling [96, 97]. Therefore, Wnt signal pathway may be a factor that contributes to the dysfunction of DCs during sepsis.

### 4.4. Epigenetic Mechanisms

Epigenetic regulation refers to external modification on gene activity without any changes in DNA sequence. Epigenetic mechanisms have been involved in the maintenance of various genes expression during embryogenesis and caner [98, 99]. In eukaryotic cells, nucleosome is the basic unit of chromatin, consisting of a short length of DNA wrapped around eight histone protein cores (duplicated in H2A, H2B, H3, and H4) [100, 101]. More and more investigators have discovered that histone modifications, including acetylation, ubiquitylation, methylation, and phosphorylation, are important epigenetic mechanisms of gene expression [101]. It is reported that maintenance of Th1/Th2 memory and gene* Il17 *expression are associated with ace acetylation and methylation of histone [102]. Histone methylation, especially for the methylation of histone H3 at lysine-4 (H3K4) and at lysine-27(H3K27), is known as a critical mechanism correlated with transcriptional activation and repression [103, 104]. Methylation at H3K4 mediated by MLL family histone methyltransferase (HMT) complex, in conjunction with several structural proteins including WD40-repeat proteins WDR5, RbBP5, and Ash2L, contributed to transcription activation [102, 105] Methylation at H3K27 is mediated by polycomb repressive complex 2 (PRC2) which contains several core components including EZH2, suppressor of Zeste 12 (SUZ12) and embryonic ectoderm development (EED) [104]. It is correlated with transcription silencing. The production of IL-12 as discussed above, an important cytokine directing Th1 immune response, was dramatically depressed in DCs from both septic patients and mice. To test if the aberrant change of IL-12 is correlated with epigenetic mechanism. Chromatin immunoprecipitation techniques were performed and data show that the reduction of IL-12 is mediated by decreasing the H3K4 trimethylation and increasing H3K27 dimethylation at* Il12p35* and* Il12p40* promoter, which result from the suppression in recruitment of MLL complex (WDR5 and RbBP5) and enhancement in recruitment of PRC2 complex (EED and SUZ12) on promoter, respectively [30]. These results indicate that epigenetic modification may be one potential mechanism of long-term immunoparalysis.

## 5. Potential Therapeutic Modulation of DC Aberrant Function

Given the central role of DCs in the immune response and survival in sepsis, it seems natural that DCs are the hopeful target for improving the aberrant immune response and prolonging the life during sepsis progression. To date many strategies for correcting the DC impaired function have been discovered, as shown in Table 1.

### 5.1. Increase the Number of DC

It has been mentioned that the loss of DCs is partly dependent on cell apoptosis, so the methods that can inhibit the apoptosis are thought to be beneficial for sepsis. IL-15 is a pluripotent cytokine that can not only coordinate the innate and adaptive immune system but also inhibit apoptosis by inducing the antiapoptotic proteins Bcl-2 and Bcl-xl in immune cells [106–108]. After the CLP operation, mice were injected s.c. with IL-15 or vehicle. Results showed that IL-15 administration significantly inhibited the apoptosis of splenic CD4, CD8, NK, and DCs induced by sepsis. During this process, IL-15 treatment increased Bcl-2 protein expression in all cells. The level of circulating IFN-*γ* was increased after IL-15 treatment, whereas both TNF-*α* and IL-6 production was decreased. Within the observation of 7 days, CLP mice treated with IL-15 had more than three-time improvement in survival compared with CLP only mice [109]. These data demonstrate that IL-15 may be a novel therapy of sepsis. Based upon the antiapoptotic molecules, TAT-Bcl-xL fusion protein and TAT-BH4 peptide were obtained and they have the ability to prevent sepsis-induced lymphocyte apoptosis, and high level of Bcl-xL improved the survival in sepsis [110]. Besides apoptosis, Fms-like tyrosine kinase-3 ligand (Flt3L) treatment was found to increase the number of CD11c^+^ DC populations by accelerating its expansion, so as to be able to reverse the endotoxin-induced tolerance [111, 112].

### 5.2. Change the DC Distribution

C5a is a potent chemoattractant among the complement products and possesses a number of functions including the modulation of cytokine and adhesion molecules expression, causing oxidant burst and granule enzymes [113–115]. C5a was reported to be excessively activated and its high expression was harmful for host during sepsis [116, 117]. After treatment with anti-C5a antibody, the IL-12^+^ DCs in peripheral blood and lymphoid nodes were decreased but were increased in peritoneal cavity in which IL-12^+^ DCs play a protection role in sepsis. Furthermore, anti-C5a antibody-treated mice had a higher survival rate than that in sham mice [118].

### 5.3. Promote DC Maturation and Increase Proinflammatory Cytokines Release

This function is the most potent in improving the immunoparalysis status in sepsis. It is known that TLR family play a critical role in the clearance of pathogen by promoting proinflammatory response. However, the activation of TLR during this process requires the interaction with coreceptor CD14 which can amplify the inflammatory signal primed by bacterial pathogen [119, 120]. So CD14 is thought to be a potential target for skewing Th1 response in sepsis. TLR2-derived peptide enhances the DC maturation by upregulation of MHCII, CD80, and CD86 expression. The peptide also increased the release of IL-12 and IFN-*γ* which are key factors for activating Th1 cell. At the same time, TGF-*β* release was inhibited. It was indicated that the TLR2-derived peptide promoted a T1 adaptive immune response and improved the status of immunosuppression [121]. In addition, the introduction of phospholipase A_2_ (PLA_2_) enhanced expression of HLA-DR, CD86, CD80, CD83, and CD40 on DCs. PLA_2_ also improved the ability of DCs to secrete IFN-*γ* when cocultured with allogeneic T cells [122]. Moreover, microRNA is also a potential target of immune modulation. Silencing of miR-142-3p which targets the IL-6 3′untranslated region significantly promoted the IL-6 expression and reduced endotoxin-induced mortality [123].

## 6. Conclusion

DCs are crucial in pathogen recognition and induction of specific immune response to protect host from the invading infection. When sepsis develops, DCs from lymphoid and nonlymphoid tissues are lost, which mostly result from the apoptosis. Several surface molecules associated with DCs maturation are changed, in which the most obvious one is HLA-DR. Upon the stimulation of external antigen or danger signal, IL-12 expression is suppressed while IL-10 production is increased, which results in the polarization of Th cell toward Th2 or Treg. During sepsis Wnt or PPAR or epigenetic-mediated mechanism may be involved (Figure 1). Several therapies that focus on improving DCs function have been shown to be able to mitigate the disease symptom. It is known that septic patients need to undergo two stages: a hyperinflammatory state and the secondary occurrence of immunosuppression. However there is no clinical parameter able to point out what the undergoing mechanism is. Therefore, specific biomarkers responsible for reflecting the immune status need to be discovered in future. Furthermore, it is imperative to find out the ideal therapeutic target that only directs to one phase without affecting the other one.

## Figures and Tables

**Table 1:** Potential therapeutic approaches for reversing DC impaired function.

Treatment	Major functions	References
IL-15	It can block sepsis-induced apoptosis of immune cells, increase the abundance of Bcl-2 while decreasing Bim and PUMA, and then increase survival.	[109]

TAT-Bcl-xL TAT-BH4	The two peptides can inhibit sepsis-induced lymphocyte apoptosis and improve survival.	[110]

Fms-like tyrosine kinase-3 ligand (Flt3L)	It can increase the numbers of DCs in spleen and reverse immunoparalysis.	[111, 112]

Anti-C5a antibody	It can prevent IL12^+^DC cells migration from the peritoneal cavity to peripheral blood and lymph nodes, thus improving survival.	[118]

TLR2-derived peptide	It can promote DC maturation and Th1 adaptive immune response.	[121]

Phospholipase A_2_ (PLA_2_)	It can promote DC maturation and increase the IFN-*γ* secretion.	[122]

Silencing of miR-142-3p	It can promote the expression of IL-6 and then reduce endotoxin-mediated mortality.	[123]
